# Exploring the path for patient organizations to participate in medical security for rare diseases

**DOI:** 10.3389/fpubh.2025.1484286

**Published:** 2025-04-02

**Authors:** Tian-tian Dong, Dong-yi Xie

**Affiliations:** School of International Pharmaceutical Business, China Pharmaceutical University, Nanjing, China

**Keywords:** rare disease, patient organization, “welfare pluralism” theory, development path, public policy

## Abstract

The participation of patient organizations in the construction of medical security systems for rare diseases is an important proposition in the theory of welfare pluralism, which indicates that individualized services and diversified connections of patient organizations can fully meet the multiple medical insurance needs of patients with rare diseases. In actual practice, however, patient organizations continue to experience institutional and structural difficulties, such as improper supervision, insufficient capacity and insufficient coordination. The theory of welfare pluralism and the developmental experience of foreign rare disease organizations have suggested that patient organizations should clarify the bridge positioning and specialized development path in the multi-subject cooperation of organizations providing medical security for rare diseases, thereby improving the efficient implementation of medical security policies for rare diseases.

## 1 Introduction

Rare diseases are a group of diseases with a very low prevalence, with the World Health Organization estimating that all rare diseases affect only 0.65 ~ 1% of the global population (1). Because China has a large population base, however, the number of patients with rare diseases in China is also large, estimated at 20 million in 2022, with the number of new patients exceeding 200,000 per year (2). Medical security that refers to the fact that patients can receive adequate medical services, including but not limited to treatment, nursing, rehabilitation, palliative care for patients with rare diseases has therefore become a social issue in China that cannot be ignored. The National Health Insurance Administration has provided important instructions on the medical security of patients with rare diseases. These instructions require continuous improvements of multi-level medical security systems for these patients, emphasizing the participation of social forces. As a result, the participation of patient organizations as representatives of patients’ interests and strength in medical security for rare diseases has become an important part of the medical security policy for rare diseases in China.

In recent years, China has established a number of rare disease organizations, including the Beijing Blood Friends Home Rare Disease Care Center, the Beijing Porcelain Doll Rare Disease Care Center, and Hyacinth Care. According to the Chen and Li (17), there were 130 rare disease patient organizations with regular activities in 2021. These organizations encourage increasing numbers of rare disease patients to help themselves and others. Patient organizations provide precise services based on the characteristics of the patients themselves and act as a bridge to mainstream healthcare services. By promoting the circulation and utilization of medical resources, these organizations can make up for the lack of government protection of patients with rare diseases, meet the actual needs of these patients in a diversified way, and provide the ultimate medical security for patients with rare diseases.

Organizations serving rare disease patients in China, however, still face challenges related to the relative age of the organization and level of expertise of the organization leader. Many organizations also have structural challenges, such as a lack of long-term growth plan, high turnover rate of leaders and members, limited number of interested medical and research professionals, and lack of public awareness of the rare condition. Welfare pluralism refers to the fact that social welfare can be shared by government departments, non-governmental organizations, the business sector, and families, and these entities provide different types of benefits to residents according to their own characteristics. At present, this theory is widely used in the study of social security issues such as medical care, education, and employment. Therefore, from the perspective of the theory of welfare pluralism, and drawing on the experience of international rare disease organizations, this study explored the paths for organizations of patients with rare diseases to participate in medical care in China.

## 2 Theoretical basis and construction of a framework for patient organizations to participate in medical security for rare diseases

### 2.1 The theory of welfare pluralism and its applicability

Welfare pluralism advocates the diversification of responsibility for social welfare, with welfare sources not being completely dependent on the state or on the market. Welfare is the product of the whole society and should be shared by all subjects, including social organizations and families. The theory of welfare pluralism was originally formulated in Britain following the economic crisis of Western countries during the 1970s (3). To reduce financial pressures and solve national crises, the government seeks to diversify the responsibility for welfare to include volunteer organizations and charities, as well as the state (4). Later, foreign researchers divided Welfare pluralism was later divided into two schools, the three-point method, which defines the sources of welfare as the state, the market and the family or civil society, emphasizing cooperation among these three sources; and the four-point method, which defines the sources of welfare as the government, the market, voluntary organizations and informal organizations (5). Both schools emphasize the responsibility of social forces in providing welfare. Volunteer organizations are thought to act as contracted agents of the state in providing welfare, with cooperative relationships between these organizations and the government (6).

Decentralization and participation are considered the core concepts of the theory of welfare pluralism. Decentralization means that power in the field of welfare should not only devolve from the central government to local governments, but also from government departments to the market and society. Participation means that welfare consumers, the private sector and non-profit organizations can all participate in decision-making and the provision of welfare services (7). In addition, the theory of welfare pluralism emphasizes that, through institutional and structural arrangements, different entities can participate in the provision of welfare services, thereby alleviating the financial pressure on the state and taking advantage of the strengths of different entities (8).

Most research on the theory of welfare pluralism has included analyses of the different levels of responsibility among subjects, focusing primarily on elder-care. These studies have lacked in-depth exploration of medical security, especially for socially vulnerable groups, including patients with rare diseases. This present study applies the theory of welfare pluralism to the issue of medical care for the patients with rare diseases. Additionally, this research explores the role of rare disease patient organizations as the key party responsible for medical security for rare disease patients. The goal of this research is to ultimately improve the medical security system for rare disease patients in China.

### 2.2 Theoretical framework for the participation of patient organizations in medical security for rare diseases

The 2018 Regulations on the Registration and Administration of Social Organizations (Draft for Solicitation of Comments) clarified the definitions and categories of social organizations, including social groups, foundations, and social service organizations. Organizationally, they are all subordinate to social organizations and some of them have obvious non-profit characteristics. Social organizations must have voluntary participation, provide public welfare, and spread public awareness, by this definition, rare disease organizations meet the responsibilities proposed in the theory of welfare pluralism. Moreover, these organizations should actively promote social welfare and assume moral responsibilities based on social values (9). From the perspective of organizational activities, the service work carried out by patient organizations for patients belongs to the category of social medical and health welfare. Patient organizations aim to help patients with rare diseases obtain fair, timely and effective medical services. This can protect the health rights and interests of patients with rare diseases. In summary, the participation of patient organizations in the construction of medical security systems for rare diseases and the theory of welfare pluralism emphasize that social organizations should be primarily responsible for social welfare. The theory of welfare pluralism can provide a theoretical basis and practical pathway for patient organizations to participate in medical security for patients with rare diseases.

**Figure 1:**
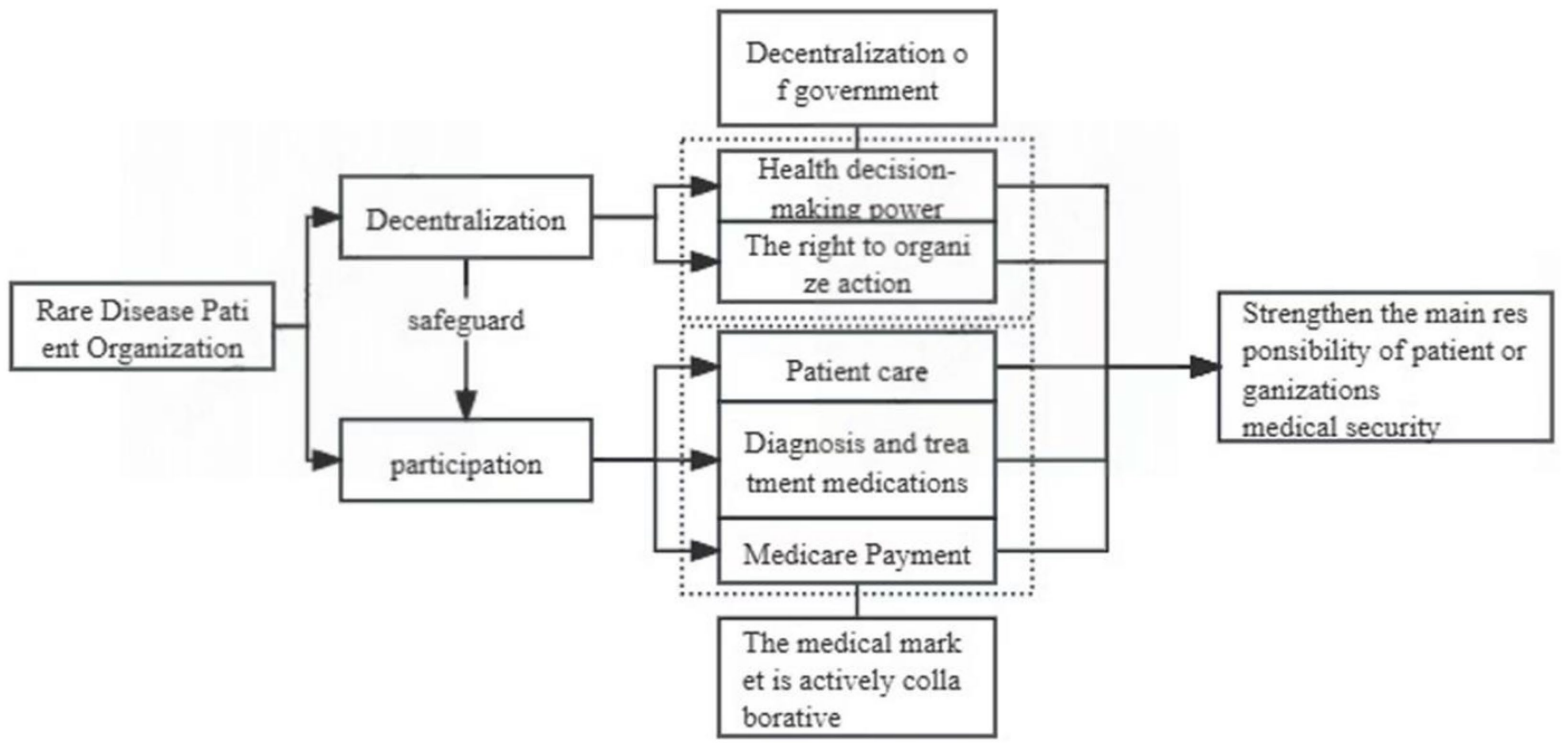
Theoretical framework for patient organizations to participate in medical security systems for patients with rare diseases.

Based on the above analysis, a theoretical framework was established for the participation of patient organizations in medical insurance for patients with rare diseases (Figure 1). Starting from the core concept of welfare pluralism, the framework strengthened the main responsibility of patient organizations in providing medical insurance for patients with rare diseases through government decentralization and active cooperation in the medical market. In terms of decentralization, the government gives patient organizations the right to make health decisions and organize actions in the medical security of rare diseases. Patient organizations can legally participate in the process of formulating rare disease protection policies and organizing publicity, fundraising and other activities, so as to improve the status of patient organizations as the body of society. Participating patient organizations can therefore interact with the medical market and participate in patient care. The decentralization of responsibility of patient organizations can provide legal protection for patient organizations to participate during the process of patient diagnosis and treatment and the exploration of medical insurance payment methods. Thus, by protecting patient rights and actively intervening in the medical treatment process, patient organizations can improve the construction of medical security systems for patients with rare diseases.

## 3 The practical dilemma of low patient participation in medical security systems for rare diseases

### 3.1 Lack of institutional and standardized guarantees

The lack of necessary institutional environments hinders the development of social organizations (10). Most patient organizations for rare diseases in China cannot meet the requirements of Chapter III of the Regulations on the Registration and Administration of Social Groups, such as the number of members in the establishment and registration, which makes it difficult for them to register in compliance. As of 2020, the success rate of rare disease patient organizations in China is less than 30% (11). Lack of legal identity leads to the lack of relevant institutional protection for these organizations, further affecting their work. These rare disease patient organizations lack normative protection, with most also lacking legal identity, making it difficult for them to obtain the relevant organizational articles in laws and regulations. The guidance and normative protection of the internal governance of their organizational structures limit their financing activities, with most having economic difficulties. A survey of the China Rare Disease Alliance found that over 80% of these organizations had raised less than 200,000 yuan in 2018, with more than 40% of these patient organizations raising no money, thus hampering their normal operation. Most legal and regulatory provisions on patient organizations are broad, only setting the requirements for annual review. In contrast, there are no specific regulatory provisions on their activities, such as financing, and the delineation of review units is vague, weakening the social credibility and authority of patient organizations.

### 3.2 Insufficient professional service capacity

Due to the lack of professional personnel in rare disease organizations, these organizations cannot provide high-quality professional services to meet the needs of patients with rare diseases, such as training on patient care activities. Because of the shortage of professional medical personnel in China, patients with rare diseases are generally cared for by their family members or recruited volunteers. But most of them are lack of the knowledge and skills required for home care. The lack of medical professionals in patient care organizations limits the ability of patients to receive scientifically valid care. Most of the sponsors and leaders of rare disease organizations in China are patients or their families, with few professional full-time staff members acting as management to formulate and participate in planned activities for these patients. At present, more than 90% of rare disease organizations with fewer than five full-time staff members in China have a name but no actual activities related to supporting patients in medical care, such as science popularization activities on disease knowledge (12). This has limited the service advantages of these patient organizations, due to lack of personnel and high turnover rate of staff. This can also limit cooperation with the government and the medical market.

### 3.3 Lack of collaboration

Due to patient psychological factors and geographical challenges, rare disease organizations in China have difficulty communicating and cooperating with other organizations. As a result, they cannot build bridges between patients and the government or the market, blocking their link to medical resources. Because rare disease organizations lack the ability to formulate policies and to communicate with other organizations. There are barriers to their involvement in rare disease policy discussion and drug development. Moreover, these organizations lack effective measures to guide patients to understand the security plan and apply for protection, resulting in policy communication barriers between the government and patients. Moreover, the collaboration of these organizations with medical institutions is not strong, leading to an imbalance in market medical resources. At present, few patient organizations are actively participating in rare disease research at medical institutions in China, with only two rare disease patient organizations in China, the SMA Care Center and the Beijing Bow Tuberous sclerosis Rare Disease Care Center, having participated in clinical trials of rare drugs. Finally, organizations for patients with rare diseases in China have shown little cooperation with international organizations for patients with rare diseases, thus blocking organizations in China from obtaining experience in medical protection for rare diseases. To date, the Kode Rare Disease Center and organizations in Taiwan are the only rare disease patient organizations in China that have joined the Association for Orphanet Patients and the International Rare Disease Research Alliance as consultants. In Hong Kong, few patient organizations cooperate with international organizations for patients with rare diseases.

## 4 Foreign organizations of patients with rare diseases

Foreign organizations for patients with rare diseases, such as the National Organization for Rare Disorders (NORD, US), and European Organization for Rare Diseases (EURODIS, Europe), are of long-standing duration and relatively complete development. These organization include many experts in rare diseases and streamlined, efficient full-time management teams, with considerable experience in technology, research, financing, and operation, providing strong support for the medical security of patients with rare diseases.

### 4.1 Multi-party forces empowering patient organizations

Most of the members of international rare disease patient organizations are authoritative institutions or individuals with outstanding abilities, with professional knowledge and considerable experience in medical protection and organizational supervision. For example, the European Organization for Rare Diseases (EURORDIS), established in 1997, is composed of representatives of members of national rare disease patient organizations of EU member states. With the human and financial support of health institutions and corporate foundations, the National Organization for Rare Diseases in the United States (NORD), founded in period of 1983 by patients, advocates, family members, healthcare providers, and policy-makers of rare disease patient organizations, successfully introduced the Orphan Drug Act. NORD has also actively explored financial incentives for the treatment of rare diseases, laying a solid foundation for the development of rare disease research. Subsequently, the board of directors of NORD, consisting of professionals from hospitals, universities and policy development institutes, has joined forces with individuals who have demonstrated outstanding leadership in serving rare disease patients to provide more comprehensive protection for rare disease patients.

### 4.2 Special care function of patient organizations

Patient care provided by foreign rare disease patient organizations has mainly focused on the psychological state of these patients, improving their quality of life and helping them better integrate into society. For example, NORD provides efficient and professional assistance in the clinical diagnosis and psychological care of these patients, with information resources shared among 31 centers of excellence for rare diseases in the United States; moreover, each of these centers can provide accurate disease diagnosis and rapid and optimal multispecialty clinical care services (13). Patients can obtain this information at NORD, and refer to this information to seek their own diagnosis and treatment, which can meet the medical needs of patients with rare diseases more quickly than ordinary diagnosis and treatment models. NORD also has psychological counseling teams within each patient organization to provide professional psychological and spiritual counseling. EURORDIS also provides special care for the treatment, rehabilitation and socialization of patients throughout the life cycle, including a contact platform for patients and regular assessment of their psychological state, with interventions provided when necessary (14).

### 4.3 Cooperation with multiple

European and American organizations of patients with rare diseases actively cooperate with their governments to provide legal protection and financial support for these patients. NORD launched the Rare Disease Advisory Councils program in 2020 and established the Rare Disease Policy Advisory Committee to give patient organizations the opportunity to make policy recommendations to national leaders. For example, they reduce the financial burden on patients by participating in amendments to the Orphan Drug Act and the Inflation Reduction Act, and ensuring that the Centers for Medicare and Medicaid Services (CMS) can negotiate the price of high-value rare drugs. In 2000, the European Medicines Agency established the Orphan Medicines Committee, whose vice-chair has been held by patient representatives (15). Moreover, the European authorities have included patient representatives as full and permanent members of the Orphan Medicines Committee with equal voting rights to allow patient organizations to provide their views on the regulation of rare medicines. The International Organization for Rare Disease Patients actively promotes cooperation with pharmaceutical companies to participate in rare disease research. The International Association on Progressive Fibrodysplasia Ossification, along with biopharmaceutical companies, developed decision-making guidelines that recommended best practice standards for interactions between patient organizations and pharmaceutical companies, ultimately having a positive impact on the development of new therapies (16).

### 4.4 Expansion of the experience of foreign rare disease patient organization to China

The development process and existing security functions of rare disease patient organizations in Europe and the United States have included improvements in the capacity of each organization with the help of the government, pharmaceutical companies and other industries. These processes have focused on strengthening the ability of these organizations to provide patient care, as well as their cooperation mechanism with various industries. This has effectively improved the participation of rare disease patient organizations in rare disease medical security, as well as promoting the development of rare disease security mechanisms. Organizations of patients with rare diseases in China can learn from the experience of these foreign organizations and explore reasonable ways to participate in medical security based on self-construction, patient care, and collaboration with foreign organizations.

## 5 Pathways for patient organizations to provide medical security for rare diseases

Based on the theory of welfare pluralism, an analysis of the dilemma associated with the development of rare disease patient organizations in China, and the experience of foreign rare disease patient organizations, this study attempted to construct a reasonable framework for the participation of rare disease patient organizations in medical security in China.

### 5.1 Strengthening policy guarantees and stimulating the autonomy of patient organizations

During the transfer of some medical security powers for rare diseases to patient organizations, the government should establish and improve laws and regulations related to the development of these organizations, thereby ensuring their legal status. The current conditions for the registration and approval of patient organizations should be appropriately relaxed by, for example, reducing the number of personnel and office space requirements. Moreover, national patient organizations should be subsidized through competitive selection, with more talented personnel and larger activity subsidies provided to local patient organizations, thereby stimulating their autonomy. The supervision of patient organizations should be strengthened, with local governments being responsible for the overall supervision of provincial rare disease patient organizations, and local civil affairs departments provided greater supervision through regularly visits, annual inspections, and reviews of tax status. The government should improve regulations as soon as possible, establish the right of rare disease patient organizations to participate in healthcare decision-making, and stipulate that patient organizations participate in decision-making hearings, thereby completing the policy participation of patient organizations.

### 5.2 Enhancement of professional capacity and cultivation of specialized patient organizations

Experienced rare disease patient organizations, such as the Beijing Pain Challenge Foundation and the Cord Rare Disease Development Center, should be encouraged to rely on the China Rare Disease Patient Organization Empowerment Project, and to pair up with developing patient organizations. Established organizations should also provide practical services and publicity, accelerate the cultivation of professional rare disease patient organizations, expand the endogenous advantages of specialization, and provide professional services to groups of patients with rare diseases. Rare disease patient organizations should strengthen cooperation with educational and scientific research institutions and medical institutions to attract medical professionals to join patient organizations. Healthcare organizations recognize the difficult situation of rare disease patients. With the help of medical institutions and professionals, rare disease patient organizations carry out professional training courses in management, education, care, and psychological counseling. Such a collaboration can not only enhance the professional skills of the organization’s practitioners, but also meet the diverse needs of patients with rare diseases in terms of care and psychology. These patient organizations should also establish personnel exchange mechanisms with the public sector, thereby optimizing the internal governance structure of patient organizations. Moreover, these organizations should establish a board of directors and a board of supervisors, thereby enhancing their social transparency and credibility, and strengthen the understanding and recognition of these organizations by the government and the medical market.

### 5.3 Clarification of the rights and responsibilities of patient organizations and improving collaborative guarantees

The most important role of patient organizations in providing medical security for patient with rare diseases is to provide connections among patients, the government and the healthcare system. Patient organizations should emphasize the advantages of resource provision, and play a role in developing and implementing policy to benefit rare disease patients. Patient organizations can actively organize patients to participate in social research on rare diseases, collect relevant data on patients’ actual needs, formulate conclusions, and assist the government in decision-making. Patient organizations can also rely on rare disease information networks to establish a unified communication platform, compile the latest policy information into a book and provide it to patients. In providing information to rare disease patients living in remote areas, these organization can use the method of door-to-door popularization of policies to help patients and their families understand policy preferences and obtain protection.

In evaluating drugs for the diagnosis and treatment of rare diseases, patient organizations should take the initiative to contact hospitals; learn and integrate information on disease diagnosis and treatment, drug acquisition, and hospitals; actively hold doctor-patient exchange meetings; recommend authoritative hospitals and doctors to patients; and solve patients’ medical needs. Patient organizations can also guide pharmaceutical companies to cooperate with patients; encourage patients to actively participate in clinical trials of rare drugs; provide feedback on drug use to pharmaceutical companies; and promote the completion of projects such as charitable donations of drugs and assistive devices; and disburse special funding from pharmaceutical companies, thereby improving patient drug use and quality of life.

### 5.4 Establishment of a demand response mechanism and improving the feedback process

The government should take the lead in setting up a working group on patient participation, consisting of participants selected by all interested parties, who will be responsible for the overall planning of patient feedback and responses from each subject. Feedback pages should be set up at key locations in the official website of the patient organization, along with patient registration channels to provide feedback on a regular basis. Organizations of patients with rare diseases should draft written documents, such as experience and tips for patients with condition, as well as unmet needs of the patient population. These documents should be submitted to the working group for processing and response, facilitating the timely adjustment of relevant decisions, with these adjustments publicized results on the organization’s official website. Organizations of patients with rare diseases should also establish a regular return visit mechanism for patients, and report on the effect of treatment and patient satisfaction and medical guidelines about the condition, that tells patients, families, and doctors about the care needed for the disease, as well as the shortcomings and prospects of patient participation, thereby assisting in the subsequent adjustment of the response mechanism and decision-making process.

## 6 Conclusion

Under the theoretical framework of welfare pluralism, the role of rare disease patient organizations in supporting the medical security of rare disease patients has been elevated to a position that can be taken seriously. This paper applies the theory of welfare pluralism to improve the quality of life of rare disease patients, and proposes to construct a rare disease patient support system with rare disease patient organizations as the core. However, the current situation of rare disease patient organizations in China is not enough to complete such work, so we put forward some new suggestions for the development of rare disease patient organizations in China by analyzing the successful practical experience of rare disease patient organizations in Europe and the United States. These recommendations include creating a favourable policy environment, providing appropriate conditions for development and building efficient working mechanisms. In these processes, the support and cooperation of the government and medical institutions is indispensable. At the same time, this is also in line with the proposition of welfare pluralism and broadens the application of welfare pluralism theory in the field of medical and health care.

One focus of this study is on the construction of a support system for patients with rare diseases from the perspective of welfare pluralism. This reflects the current challenges of isolation, lack of collaboration, and fragmentation of current rare disease patient populations. The recommendations in this study promote a more sustainable developmental framework for rare disease patient organizations to combat this fragmentation and improve quality of life of patients. However, there are still certain limitations to how strategies can be integrated with each other, and deeper exploration is needed. In the next step of research, it is necessary to strengthen the research in three aspects:

First, at the theoretical level, the paper focuses on using welfare pluralism to emphasize the dominant position of rare disease patient organizations in patient support. But it does not involve much in the functions of other subjects, such as how researchers should actively incorporate rare disease patient organizations into disease clinical research. Follow-up research on patients with rare diseases can focus on clarifying the functions of each subject and refining the collaborative governance mechanism between the subjects.

Second, at the practical level, it is necessary to create a clear plan for funding to help rare disease patient organizations grow, develop, and carry out awareness and research initiatives. Rare disease patient advocacy groups are often tasked with broad responsibilities including building community, sharing research opportunities, disseminating research advances and popularizing science, advocating for policy change, and more. Lack of funding threatens the sustainability and influence of these organizations.

Third, at the process level, there is a need to establish a link and dynamic adjustment mechanism for the whole life cycle. Follow-up research on implementation and response involving cross-departmental, cross-field, multi-subject and multi-link is an important direction in the future.

## References

[1] Lu J Pan X. Discuss the research status and future of rare diseases in China. Chin Trop Med. (2023) 23:109–14.

[2] Li C Fang H. (2022). Caring for more than 20 million rare disease patients: China accelerates the exploration of the “Chinese model” of diagnosis and treatment and protection of rare diseases. [2023-08-81]. Available online at: https://www.gov.cn/xinwen/2022-11/03/content_5724188.htm

[3] An S. From fragmentation to plurality and synergy: An analysis of the main responsibility of rural long-term care supply from the perspective of welfare pluralism theory. J Henan Univ Eng. (2023) 38:49–54.

[4] Gilbert N. Welfare pluralism and social policy In: Midgley J Tracy MB Livermore M, editors. Handbook of social policy. Thousand Oaks, CA: Sage (2000). 61.

[5] John O. On welfare pluralism, social policy and the contribution of sociology: revisiting Robert pinker. Front Sociol. (2023) 8:1076750–07. doi: 10.3389/fsoc.2023.1076750, PMID: 37139226 PMC10149760

[6] Lin M Wang Z. Nonprofit research from a diverse perspective on benefits. Soc Sci Res. (2001) 6:103–7.

[7] Yue Z. An analysis of medicare policy from the perspective of welfare pluralism. Public Adm Rev. (2009) 2:37–51+202.

[8] Chaney P. Multi-level systems and the electoral politics of welfare pluralism: exploring third-sector policy in UK Westminster and regional elections 1945-2011. Volunt Int J Volunt Nonprofit Org. (2014) 25:585–611. doi: 10.1007/s11266-013-9354-9

[9] Chen Y Pang F. Research on the subject composition and functional relationship of welfare pluralism. Jianghai J. (2020) 1:88–95.

[10] Dou Y Jin J. The profit-seeking logic of a non-profit organization: take an organization of patients with a genetic metabolic disease as an example. Sociol Rev. (2022) 10:212–29.

[11] Zhang X Dong D. China rare disease comprehensive social survey. Beijing: People’s Medical Publishing House (2020).

[12] Huang R. Current status of rare diseases in China. Cord Center for Rare Diseases: Beijing (2018).

[13] NORD Rare disease centers of excellence. 2023-06-28. Find a center [2023-07-08]. Available online at: https://rarediseases.org/rare-disease-centers-of-excellence/

[14] Hollak CE Biegstraaten M Baumgartner MR Belmatoug N Bembi B Bosch A. Position statement on the role of healthcare professionals, patient organizations and industry in European reference networks. Orphanet J Rare Dis. (2016) 11:1–7. doi: 10.1186/s13023-016-0383-5, PMID: 26809514 PMC4727340

[15] Mavris M Le Cam Y. Involvement of patient organizations in research and development of orphan drugs for rare diseases in Europe. Molecular Syndromol. (2012) 3:237–43. doi: 10.1159/000342758, PMID: 23293582 PMC3531929

[16] Stein S Bogard E Boice N Fernandez V Field T Gilstrap A. Principles for interactions with biopharmaceutical companies: the development of guidelines for patient advocacy organizations in the field of rare diseases. Orphanet J Rare Dis. (2018) 13:18. doi: 10.1186/s13023-018-0761-2, PMID: 29357903 PMC5778794

[17] Chen Y Li Y. China rare disease comprehensive report. (2021) [2023-08-81]. Available online at: http://www.diagnoschina.com/research/detail/nid-83, PMID: 23293582

